# Comparative evaluation of volumetric changes of three different retrograde calcium silicate materials placed under different pH condititions

**DOI:** 10.1186/s12903-020-01325-x

**Published:** 2020-11-19

**Authors:** So Yeon Kwon, Min-Seock Seo

**Affiliations:** Department of Conservative Dentistry, Wonkwang University Daejeon Dental Hospital, 77 Dunsan-Ro, Seo-Gu, Daejeon, 35233 Republic of Korea

**Keywords:** Calcium silicate cement, Human incisors, Retrofilling, Acidic setting condition, Micro-computed tomogrphy

## Abstract

**Background:**

The present study aimed to compare the volumetric changes of three calcium silicate cements after retrofilling and placing under different pH conditions via micro-computed tomography (micro-CT) scan.

**Methods:**

Forty-two extracted human single-rooted teeth were randomly assigned to three groups according to the retrofilling materials used (Biodentine, Endocem MTA, and ProRoot MTA). Each group was divided into two subgroups according to the setting condition. The teeth in one group were immersed in normal saline for 5 days at room temperature, and the teeth in the other group were immersed in butyric acid (pH = 5.4) for 5 days at room temperature. The volume ratios of the retrofilling material were calculated via micro-CT imaging.

**Results:**

The volume ratios of the Biodentine and Endocem MTA groups were significantly different between the two setting environment, and these groups had significantly lower filled volume ratio (Vf, %) in the acidic environment than in the saline environment (pH = 5.4). Meanwhile, the volume ratio of the ProRoot MTA group did not significantly differ between the two setting environments. All materials under the acidic setting condition had relative radiolucency in the area in contact with the acidic solution.

**Conclusion:**

The Vf ratio of the Biodentine and Endocem MTA cements was significantly lower in the acidic environment than in the saline environment. No statistically significant difference was observed in the Vf ratio of ProRoot MTA between the two setting environments.

## Background

Mineral trioxide aggregate (MTA) is a calcium silicate-based cement commonly used in endodontic treatment, such as perforation repair or root end filling in apical surgery. It has several advantages in terms of biocompatibility, sealing ability, and setting ability in a hydrophilic condition. In terms of advantages, particularly during surgeries, MTA can reduce micro-leakage at the end of the apex when used as a retrofilling material, act as a stable barrier, and improve the healing ability of the periapical tissue with high biocompatibility [1–3].

Nevertheless, MTA has some disadvantages, which typically include difficulty in handling and slow setting time [4]. Challenges in handling make it difficult to fill MTA into the cavity [5]. In apical surgery, a long setting time makes it difficult to confirm complete setting of MTA. In addition, washout of the unset MTA can cause relapse of the periapical lesion [6].

Therefore, numerous studies about materials that can rapidly set and are easy to handle have been conducted, and the use of various materials has been introduced recently. Endocem MTA (Maruchi, Wonju, Korea) is a pozzolan cement, which has a quick setting time, excellent sealing capability, and outstanding biocompatibility and is easy to handle [7]. Thus, the outcomes are consistent. Choi et al. have reported that the biocompatibility and osteogenicity of Endocem MTA cement are similar to those of ProRoot MTA (Dentsply. Caulk, Milford, DE, USA), and Endocem MTA had a higher resistance to washout than ProRoot MTA [7]. The other material is Biodentine (Septodont, Saint Maur des Fausses, France), which is a calcium silicate cement designed as a dentine replacement material. It is available in the form of a capsule containing the ideal ratio of its powder and liquid and is mixed using trituration. Characteristics, such as fast setting time (10–12 min), excellent sealing properties and ease of handling, make Biodentine a suitable retrofilling material [8, 9].

In surgical endodontic treatment, the marginal sealing ability of the retrofilling material used is important to prevent the growth of bacteria. Gap formation, low dimensional stability, or loss of material can cause the re-growth of bacteria [10]. Kim et al. have reported that ProRoot MTA had a higher gap formation than Endocem MTA after it was immersed in saline when used as a retrofilling material [11]. However, the condition of the surrounding tissue after the apical surgery may have lower pH levels due to infection and inflammation [12, 13]. In previous studies, when the inflammatory process in the adjacent tissue is controlled with endodontic treatment, the pH returns to slightly alkaline (pH = 7.4) within 7 days [13] or less [14]. As a result, the setting process of the retrofilling material may be exposed to an acidic environment in inflammatory conditions for at least 5 days.

Some experiments that involved setting retrofilling materials in acidic environments were conducted. Aksel et al. have reported that the storage in acidic condition does not affect the surface level and vertical dimension of ProRoot MTA and Biodentine [15]. By contrast, Ashofteh et al. have reported that the surface microhardness of ProRoot MTA and Endocem MTA was significantly reduced with exposure to butyric acid compared with phosphate-buffered saline (PBS) [16]. Tian et al. have found that ProRoot MTA can release a higher amount of Si and Ca ions when exposed to acidic conditions compared with PBS [17]. Moreover, some studies have assessed the release of ions and the microhardness of the surface set in an acidic condition. However, no study has evaluated the dimensional stability of different calcium silicate cements set in an acidic environment.

Thus, the present study aimed to compare the volumetric changes after retrofilling using three calcium silicate cements placed under different pH levels via micro-computed tomography (micro-CT) scan. Butyric acid was used to create an acidic solution (pH = 5.4), and phosphate-buffered PBS was utilized as the control.

## Methods

### Sample preparation

The study protocol was approved by the institutional review board of Wonkwang Dental University Daejeon Hospital (W1905/001-001). Forty-two extracted human single-rooted teeth with similar sizes were collected and stored in PBS (PBS 3813; Sigma-Aldrich, St Louis, MO, USA) until preparation. Under a dental microscope (Carl Zeiss surgical GmbH; Carl Zeiss, Gottingen, Germany), any teeth with cracks or fractured apex were excluded from the experiment. The teeth were randomly assigned to three groups according to the retrofilling materials used (n = 14). The retrofilling materials used in this study were Biodentine (Septodont), Endocem MTA (Maruchi), and ProRoot MTA (Dentsply).

To standardize the working length, the crowns of 42 single-rooted teeth were removed and the single roots with similar sized were obtained. The canals were instrumented with ProTaper Next (DentsplyMaillefer, Ballaigues, Switzerland) to a master apical size of #30 (F3) in a crown-down motion and 0.5 mm short of the apical foramen. Irrigation was performed in between every shaping motion using 1.5% sodium hypochlorite. After drying the canal with a paper point, they were obturated with gutta-percha and AH plus sealer (Dentsply) using the continuous wave technique (#30/06 GP cone + Obtura II Max System; Obtura Spartan, Fenton, MI, USA). Then, the root tips were resected 3 mm perpendicular to the longitudinal axis of the roots with diamond disc. The root-end cavity was prepared with carbide bur (SS White; FG 245, Lakewood, NJ, USA) and with the ultrasonic retropreparation diamond tip (Sybron Endo; BK3-R, Glendora, CA, USA) with distilled water to create class I cavity and a 3-mm depth with parallel walls as reproducible as possible. The bur was replaced with each preparation. To remove smear layer and clean the cavity, 37% phosphoric acid etching was done for 15 s in every cavity.

Three retrofilling materials were mixed and applied according to the manufacturers’ instructions [Powder: liquid ratio = Endocem MTA (300 mg/0.12 cc), ProRoot MTA (3:1)]. To mix Biodentine, five drops of the liquid supplied by the manufacturer were placed in the capsule with powder. Each group of specimens (n = 14) was divided into two subgroups according to the immersion medium. All samples were let stand for 5 min after filling in a dry environment and at room temperature, and then they were immersed in each solution. One subgroup was immersed in normal saline for 5 days at room temperature and the other subgroup in 1 mmol/L of butyric acid (pH = 5.4) for 5 days at room temperature. A single operator (a resident in the endodontic department for 2 years) performed all the procedures.

### Evaluation via micro-CT scan

To investigate the volumetric change, samples were examined using micro-CT imaging (Sky-Scan 1172TM, Skyscan, Kontich, Belgium). The volume ratios of the retrofilling materials were calculated via micro-CT scan up to a 3-mm level from the apex. The gap and volume of each of the filling materials were measured with an X-ray source voltage of 60 kV, beam current of 167 μA, 0.5-mm thick Al filter, rotation step of 0.4°, pixel size of 6 μm, and exposure time of 440 ms. After performing micro-CT scan, two software programs (NRecon™ and CTvox™, Skyscan, Kontich, Belgium) were used to reconstruct the two- dimensional (2D) images of the samples and to measure the filled volume (Vf) ratio (%) of the retrofilling materials. Micro-CT scan and image reconstruction analysis were conducted by one technician. For quantitative analysis of the images, CT-analyzerTM (Skyscan) was used.

### Vf ratio = filled material volume (Vm)/total prepared volume (Vt) × 100 (%)

A larger Vf value indicated a less gap formation of the material. Vf ratio of each samples were evaluated at 1-mm level and 3-mm level from root-end.

### Statistical analysis

The Kruskal–Wallis test was used to determine the statistical difference between different materials, and the Wilcoxon-signed rank test was used to compare different setting conditions (saline and acidic) using the Statistical Package for the Social Sciences software version 20 (SPSS Inc, Chicago, IL, USA), and significance level was 95%.

## Result

The median of Vf (%) values of the three calcium silicate cements are shown in Table 1. The Vf (%) of the Biodentine and Endocem MTA groups was significantly lower in the acidic environment than in the saline environment (*P* < 0.05). Meanwhile, that of the ProRoot MTA group did not significantly differ between the two setting environment (*P* > 0.05). The Vf (%) of the three materials (n = 14), did not significantly differ (*P* > 0.05).

**Table 1:** Median values of filled volume ratios of the 3 calcium silicate cements

Parameters	Biodentine median (25%/75%)		EndoCem MTA median (25%/75%)		ProRoot MTA median (25%/75%)	
	Saline Sol	Acidic Sol	Saline Sol	Acidic Sol	Saline Sol	Acidic Sol
Total Vol (mm^3^)	5.31 (5.21/5.62)	5.11 (4.72/5.33)	5.15 (4.88/5.28)	5.48 (5.31/5.79)	5.42 (5.21/5.58)	5.12 (4.99/5.23)
Filling material Vol (mm^3^)	5.17 (4.95/5.47)	4.28 (4.24/4.79)	5.03 (4.67/5.18)	4.86 (4.76/5.26)	5.1(4.97 /5.36)	4.69 (4.54/4.80)
% Vol (%)	97.52 (96.81/97.83)	89.46* (83.31/90.29)	97.70 (95.76/98.30)	88.58* (88.19/92.33)	94.68 (93.85/95.98)	91.24 (88.77/93.07)

%Vol (filled volume ratio-Vf) showed no significant statistical difference among 3 groups (*P* > 0.05)

*Statistically significant difference with saline solution (*P* < 0.05)

**Fig. 1:**
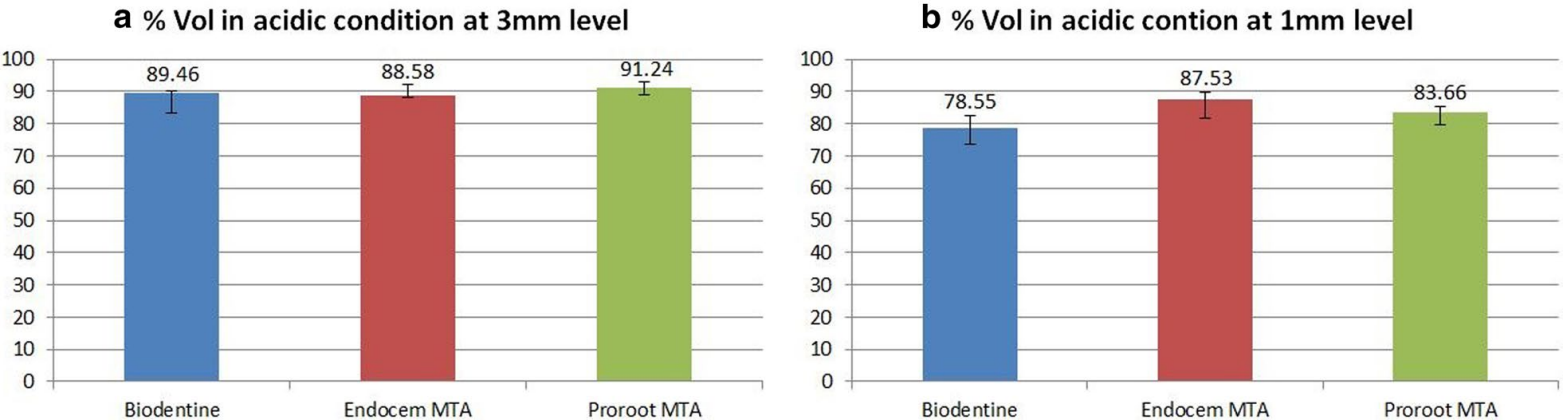
The comparison of the median filled volume ratio (%) of 3 materials set in acidic condition (n = 7, Fig. 2a shows a comparison of Vf (%) values upto 3 mm level and 2B upto 1 mm level)

In an acidic environment, the Vf (%) of the three materials (n = 7) did not significantly differ at a 3-mm level (Fig. 1a, *P* > 0.05). In addition, no significant difference was observed in the three materials in terms of Vf (%) at a 1-mm level from the apex (Fig. 1b, *P* > 0.05).

**Fig. 2.**
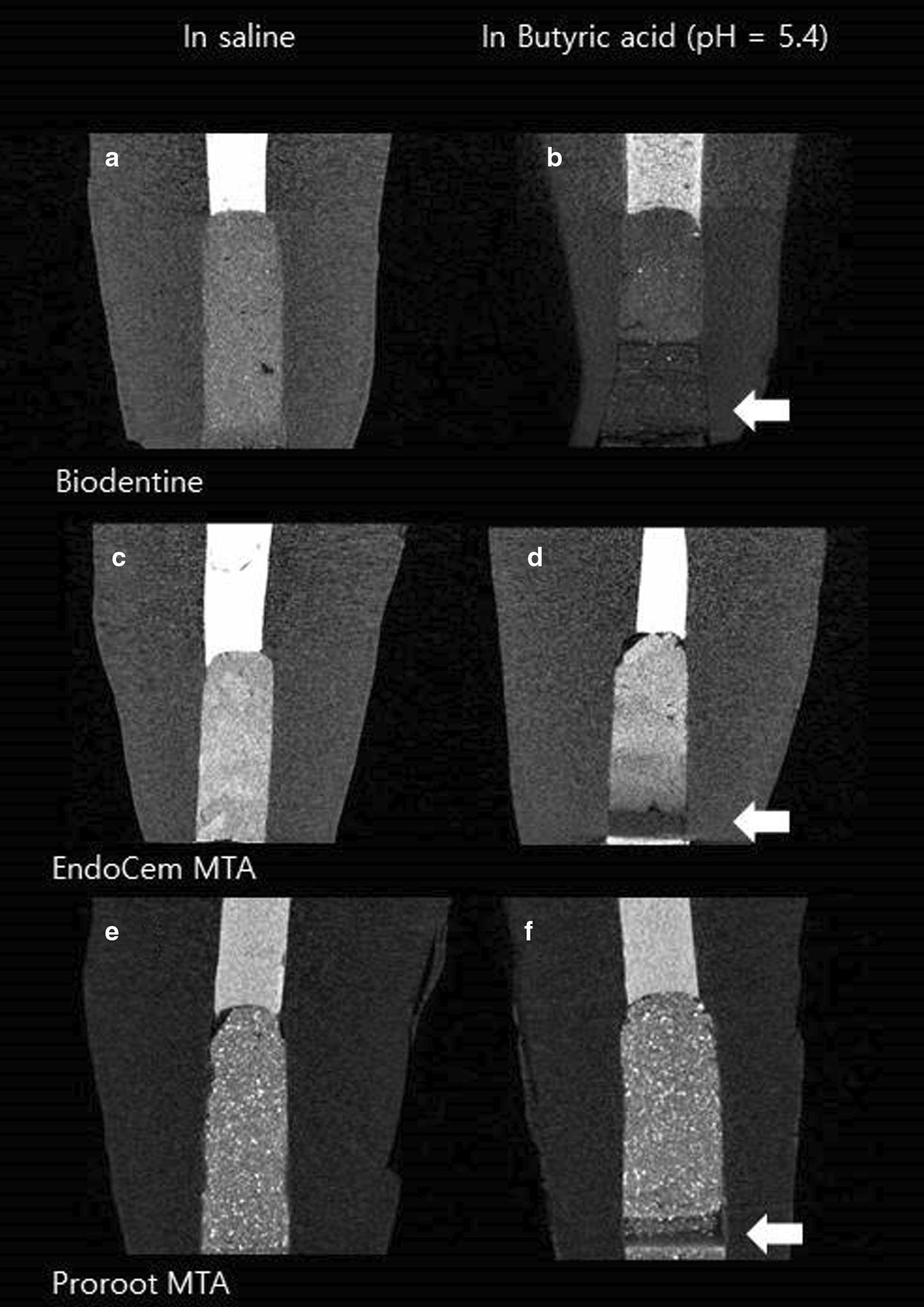
2D reconstruction of retrofilling materials in different setting condition. **a** Biodentine in saline, **b** biodentine in butyric acid, **c** EndoCem MTA in saline, **d** EndoCem MTA in butyric acid, **e** ProRoot MTA in saline, **f** ProRoot MTA in butyric acid. All materials in acidic setting condition (**b**, **d**, **f**) showed relative radiolucency in contact area with acidic solution (white arrows)

The images of the all the materials in acidic conditions showed a noticeable radiolucency at the site where it had contact with the acidic solution (Fig. 2).

## Discussion

To establish an acidic environment, this study used butyric acid, which is one of the by-products of the metabolism of anaerobic bacteria, the dominant bacteria in endodontic infections. Therefore, to simulate infectious conditions in laboratory studies, the use of butyric acid has been recommended [12, 18, 19]. PBS is a simulated tissue fluid containing phosphate that can be used to mimic normal in vivo conditions in laboratory studies [20].

Among the three materials used in the current study, Biodentine had the largest significant difference in volume ratio based on the setting condition. Therefore, this material is most affected by the acidic environment during setting process. The relatively rapid setting time of Biodentine (9–12 min) is attributed to calcium chloride, the accelerator in the liquid [21]. Initial contraction occurs at the beginning of setting. However, delayed expansion occurs after. The set material releases calcium ions into the aqueous solution. Setting continues for at least 14 days with the exchange of ions in the surrounding environment [22, 23]. In this study, the materials were immersed in acidic solution (butyric acid, pH = 5.4) for 5 days. The acidic environment might have influenced the initial contraction or affected the setting process with ion exchange in the surrounding environment, which lasted for 14 days. Aksel et al. have reported that the different effects of acidic and neutral pH levels on the properties of the materials might be correlated to the inhibition of the setting reaction [15], which may lead to the fast dissolution of the materials in an acidic environment. In relation to this reason, the solubility of the material may impair the dimensional expansion by preventing the accumulation of hydroxyapatite on the material surface [24]. Similarly, Grench et al. have reported that Biodentine has a higher wash-out tendency, with the loss of materials upon contact with blood and other fluids [9]. Moreover, Agrafioti et al. have shown that ProRoot MTA had hexagonal crystal in scanning electron microscope (SEM) after it was immersed in citric acid for 3 months, whereas Biodentine had smooth spheroidal crystal [25]. They have concluded that these structural changes in hydroxyapatite in an acidic condition may affect the solubility and porosity formation of Biodentine. In addition, Namazikhah et al. have reported that when the environment is more acidic, the setting MTA was more porous [14]. Considering the result of this study, it should be considered clinically that the volume ratio of the materials immersed in acidic solution was significantly lower than that immersed in normal saline, and it was most remarkable in Biodentine.

When comparing the Vf (%) values of the three materials, all materials had similar volume values. By conducting experiments on in-vitro settings, the handling of related variables was reduced. During the experiment, Biodentine had a similar operability to that of packable composite resin, and it was easy to pack. Endocem MTA is less viscous and easier to pack, and the texture is extremely fine and has a mud-like consistency [11]. ProRoot MTA had high adhesion as it adhered well to the instrument, and it came out even after it was packed into the cavity. Thus, the material will be more difficult to operate than the other two materials in high-level procedures, including periapical surgery.

Kim et al. have reported that ProRoot MTA had a higher gap formation than Endocem MTA when it was used as a retrofilling material in vitro [11]. Moreover, the superior consistency of Endocem MTA established a lower gap formation than ProRoot MTA. Choi et al. have found that Endocem MTA set significantly faster and was more resistant to washout than ProRoot MTA [7]. In our study, Endocem MTA had a higher Vf ratio than ProRoot MTA. However, the difference was not statistically significant. Although all the materials formed 3-mm cavities and operated according to the manufacturer’s instructions in vitro, the results of previous studies were different from ours. The difference may be attributed to the technique sensitivity during MTA manipulation. Thus, differences can be observed based on who conducted the experiments.

The images of all the setting materials in acidic conditions had a noticeable radiolucency in the area in contact with the acidic solution (Fig. 2). When calcium silicate cements set, they undergo ion exchange with the environment. Tian et al. have reported that exposure to an acidic environment enhanced the release of Si and Ca ions from ProRoot MTA and reduced the apatite formation capacities of this material [17]. Moreover, they used butyric acid with pH 5.4 in this study. Loranzo et al. have reported that Endocem MTA had increased calcium ion release after it was immersed in acid solution for 7 days [26]. Moreover, they have found prismatic crystalline structures on the surfaces of Endocem MTA after it was immersed in acidic solution. These findings are consistent with those of previous studies that reported about the presence of cubic, prismatic, and needle-like crystalline structures in bioactive cements exposed to blood, PBS, and butyric acid, respectively [16, 27, 28]. Thus, during these radiolucent phases, it is supposed that a higher ionic releases might occur in calcium silicate cements when set in an acidic environment than in saline, and these ions may contain radiopaque ions.

This experiment had some limitations. First, the size of the cavities formed were not exactly similar. However, the difference was not significant. We attempted to simulate a clinical situation using real human teeth. However, there was a difference in size, and when the cavity was larger, it was easier to fill the materials. Second, some surface areas where the radiolucency was observed were not assessed. Thus, further studies that analyze the surface areas using EDX, SEM, or micro-hardness test for the evaluation of actual leakage must be conducted.

## Conclusion

The Vf ratio of Biodentine and Endocem MTA was significantly lower in the acidic environment than in the saline environment. No significant difference was observed in the Vf ratio of ProRoot MTA between the two setting environment. Based on the comparison of Vf (%) in an acidic environment (n = 7), the Vf (%) of the three materials did not show statistically significant difference.

## Data Availability

The datasets used and analyzed during the study are available from the corresponding author on reasonable request.

## Abbreviations

MTA: Mineral trioxide aggregate
SEM: Scanning electron microscope
PBS: Phosphate-buffered saline
CT: Computed tomography
Vf: Filled volume
